# Exploring binaural hearing in gerbils (*Meriones unguiculatus*) using virtual headphones

**DOI:** 10.1371/journal.pone.0175142

**Published:** 2017-04-10

**Authors:** Sandra Tolnai, Rainer Beutelmann, Georg M. Klump

**Affiliations:** Cluster of Excellence “Hearing4all”, Animal Physiology and Behavior Group, Department of Neuroscience, School of Medicine and Health Sciences, Carl von Ossietzky University of Oldenburg, Oldenburg, Germany; Universidad de Salamanca, SPAIN

## Abstract

The Mongolian gerbil (*Meriones unguiculatus*) has become a key species in investigations of the neural processing of sound localization cues in mammals. While its sound localization has been tested extensively under free-field stimulation, many neurophysiological studies use headphones to present signals with binaural localization cues. The gerbil's behavioral sensitivity to binaural cues, however, is unknown for the lack of appropriate stimulation paradigms in awake behaving gerbils. We close this gap in knowledge by mimicking a headphone stimulation; we use free-field loudspeakers and apply cross-talk cancellation techniques to present pure tones with binaural cues via “virtual headphones” to gerbils trained in a sound localization task. All gerbils were able to lateralize sounds depending on the interaural time or level difference (ITD and ILD, respectively). For ITD stimuli, reliable responses were seen for frequencies ≤2.9 kHz, the highest frequency tested with ITD stimuli. ITD sensitivity was frequency-dependent with the highest sensitivity observed at 1 kHz. For stimuli with ITD outside the gerbil's physiological range, responses were cyclic indicating the use of phase information when lateralizing narrow-band sounds. For ILD stimuli, reliable responses were obtained for frequencies ≥2 kHz. The comparison of ITD and ILD thresholds with ITD and ILD thresholds derived from gerbils’ free-field performance suggests that ongoing ITD information is the main cue for sound localization at frequencies <2 kHz. At 2 kHz, ITD and ILD cues are likely used in a complementary way. Verification of the use of the virtual headphones suggests that they can serve as a suitable substitute for conventional headphones particularly at frequencies ≤2 kHz.

## Introduction

The processing of information carried by sounds arriving at the two ears is integral to many species' everyday tasks. These binaural cues comprise minute differences in arrival time, intensity, or spectral content and allow for competences such as localizing sounds, and separating sources in complex acoustic environments. A prominent animal model to investigate the processing of binaural cues is the Mongolian gerbil [1–5]. Because of its good low frequency hearing that parallels that of humans [6], studying sound localization and the processing of interaural time differences (ITDs) in this species has received particular attention [1,5,7–12]. Though the gerbil's sound localization ability has been investigated extensively by a number of laboratories [7,9–11], its behavioral sensitivity to ITDs and interaural level differences (ILDs), the major cues for sound localization in the azimuthal plane, remains unknown. So far, the gerbil's ITD and ILD sensitivity has only been inferred from acoustic measurements [13] and derived from its sound localization performance using free-field stimuli that favor the use of either interaural cue [9–11]. Knowledge of the behavioral sensitivities of gerbils to ITDs and ILDs will pose an important step towards interpreting findings from physiological studies.

The largest body of work on sensitivity to ITDs and ILDs exists for the human species [14–18]. While in humans the use of headphones to test ITD and ILD sensitivities is an unproblematic procedure, providing sounds via headphones to non-human animal species for determining the behavioral sensitivity to ITDs and ILDs generally requires more elaborate approaches including surgical intervention [19–28].

Here, we investigated gerbils' behavioral sensitivity to ITDs and ILDs using “virtual headphones”, a technique that allows measuring binaural perception by presenting tones dichotically using two free-field loudspeakers and applying cross-talk cancellation techniques to stimulate the ears separately [29]. The animals were trained in a one-interval two-alternative forced choice task using positive reinforcement. We first used sounds presented in the free-field. The animals learned to approach the left side of a Y-shaped platform when hearing a sound from the left and to approach the right side when hearing a sound from the right. Thus, animals were forced to use directional information contained in the stimuli to receive a food reward. Using the virtual headphones, we then determined gerbils' thresholds for ITDs and ILDs for a range of frequencies and compared those to ITD and ILD thresholds that we inferred from the animals' performance when localizing tones presented in the free-field.

## Material and methods

### Subjects

Six adult, agouti-colored, male Mongolian gerbils (*Meriones unguiculatus*) were tested in the experiment. They were bred and raised in the animal facilities of the University of Oldenburg, Germany, and originated from animals obtained at Charles River laboratories. The animals were housed in pairs in cages provided with litter and nesting material. They had unrestricted access to water but were food-restricted during testing. Their body weight was monitored daily during testing or weekly when they had free access to food. During testing, they weighed approximately 70 to 85 g. Food rewards during the experiments were 10 mg custom-made pellets. To keep the animals' body weights roughly constant during testing, food was supplemented if necessary. At the beginning of a pilot study using similar stimuli as in the current experiments, the animals were 11 to 17 weeks old. Initial training took around three weeks. Animals were 11 to 17 months old when the data presented here were collected. Data collection lasted approximately 6 months. The care and treatment of the animals were approved by the Niedersächsisches Landesamt für Verbraucherschutz und Lebensmittelsicherheit (LAVES), Lower Saxony, Germany.

### Experimental setup

Experiments were run in a sound-attenuating booth (IAC, Industrial Acoustics Company, 1203-A) lined with sound-absorbing acoustic foam (PLANO 50/0 covered with PYRAMIDE 100/100 willtec; *Seyboth & Co*.). The reverberation time *T*60 of a broadband white noise measured with a microphone positioned in the typical position of the animal’s head was 14 ms. Experiments were run in darkness to reduce the possibility of animals using visual landmarks [30]. The only light-emitting sources were green and red light-emitting diodes indicating the functioning of feeders and light-barriers. The illuminance in the booth was <0.01 lux.

**Fig 1:**
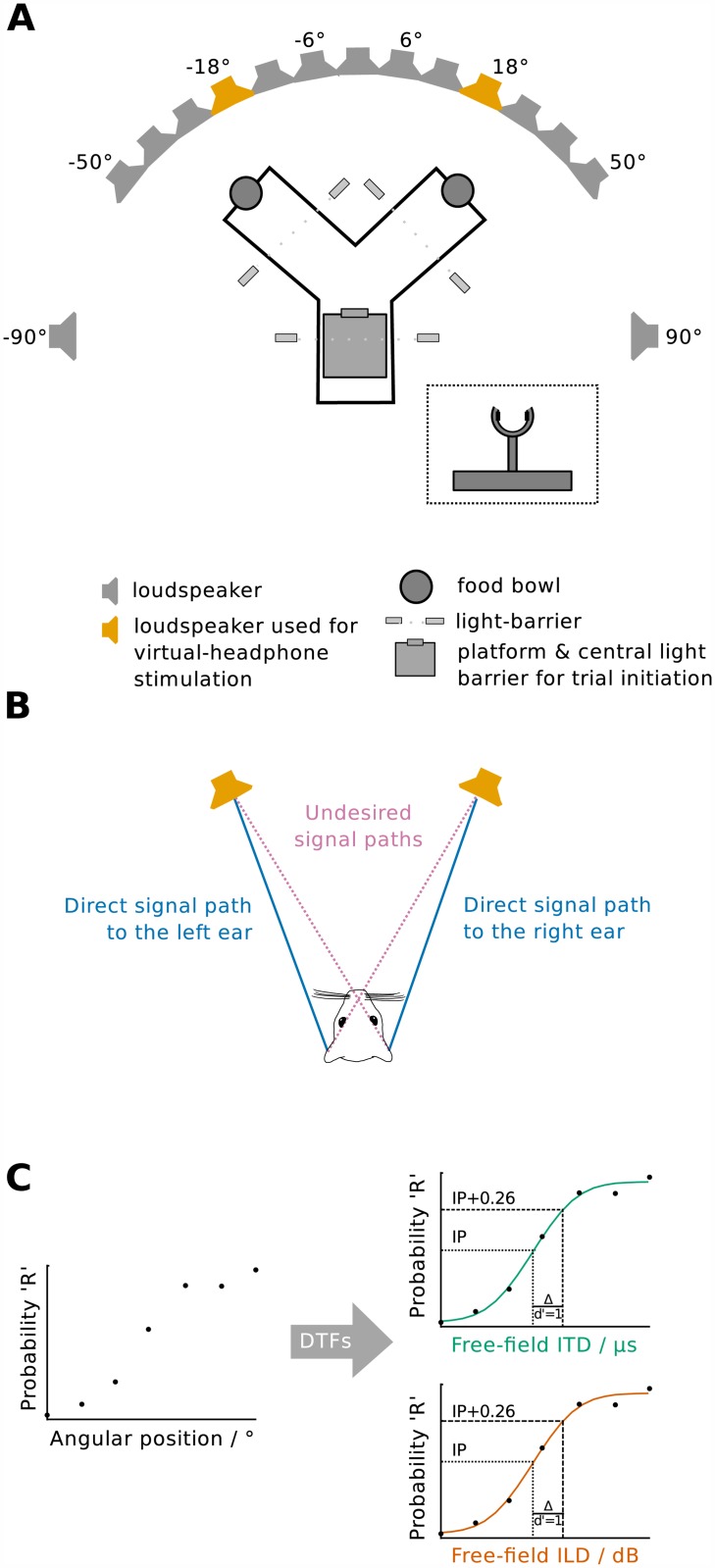
Methods and procedures. **(A)** Schematic of the experimental setup. Free-field stimuli were presented from a subset of an array of 15 loudspeakers distributed between -90° and 90° (gray and orange). Virtual-headphone stimuli were presented from the loudspeakers positioned at ±18° (orange). Animals moved on a Y-shaped platform and initiated trials by disrupting a light-barrier in a half-ring-shaped poke-hole (inset) with their nose. Animals' movements and responses were monitored by further light-barriers. Correct responses were rewarded by dispensing food rewards into food bowls from dispensers (not shown) fixed on the ceiling of the sound-attenuated booth. **(B)** Sketch illustrating the generation of virtual-headphone stimuli using cross-talk cancellation. The undesired signal paths (pink) between the loudspeakers and the respective contralateral ears are eliminated by destructive interference in the ears resulting in only the direct signal paths (blue) remaining present at the ears. **(C)** The free-field performance measured as probability of an approach to the right was transformed to free-field interaural level difference (ILD) and free-field interaural time difference (ITD) performance by extracting ITD and ILD values from the directional transfer functions (DTFs) obtained for the angular positions tested. Thresholds were then calculated by fitting a cumulative normal distribution function (green and red lines) to the raw data (circles) and determining the difference in ITD or ILD at the inflection point of the function (IP, dotted line) and 0.26 above the inflection point (IP+0.26, dashed line), thus corresponding to a d’-value of 1.

The gerbils were tested on a Y-shaped mesh platform (5 mm opening) (Fig 1A) located ~1 m above the ground and monitored by an infrared camera (Conrad Electronics). At the center of the base of the Y, a small pedestal (9.5 × 9 × 2 cm) was positioned equipped with a light barrier detecting the animal’s mounting or dismounting and a half-ring-shaped poke-hole at nose-height also equipped with a light barrier. The arms of the Y were equipped with a feeding bowl at the end and light barriers to register the animals' responses. Food was dispensed through plastic tubes from custom-built feeders mounted 50 cm above the Y-shaped platform. Fourteen loudspeakers (Vifa XT 300 K/4) were mounted on a semicircle 62 cm from the center of the half-ring-shaped poke-hole at ear height covering positions between -90° (far left) to +90° (far right).

### Free-field stimuli

Stimuli were pure tones with frequencies of 750, 1000, 1250, 2000, 2400, 3000, 4000 or 6000 kHz. All stimuli were 125 ms in duration including 25 ms Hann ramps and presented at a mean level of 60 dB sound pressure level (SPL) randomly roved by ±3 dB. Stimuli were presented from the loudspeakers to the left and to the right of the midline: ±6°, ±12°, ±30°, and ±50° and, for training only, from ±90° (Fig 1A). Negative angles denote positions to the left of the midline. The outputs of the loudspeakers were spectrally equalized between 350 Hz and 23 kHz using individual 256th order finite impulse response filters for each loudspeaker. The stimuli were computer-generated by custom-made software implemented on a Linux PC, produced by a Hammerfall DSP (Multiface II, RME; sampling frequency 48 kHz), passed through a digital signal processor for filtering (Tucker-Davis Technologies RP2.1), amplified (Rotel, High Current 8 Channel Power Amplifier RMB-1048), and distributed to the loudspeakers by an electronic multiplexer (Tucker-Davis Technologies PM2R).

### Virtual-headphone stimuli

Stimuli were pure tones, 125 ms in duration including 25 ms Hann ramps. ITD discrimination was tested at stimulus frequencies of 750, 1000, 1250, 2000, 2400, 2673, and 2900 Hz. The ITD was applied by delaying the stimulus in one channel but ramping the stimuli in both channels simultaneously resulting in stimuli with only ongoing but no onset ITD cues. Negative ITDs denote stimuli with leading left signal. Absolute ITDs tested ranged between 0 to 600 μs. ILD discrimination was tested at 2000, 4000, and 6000 Hz. Negative ILDs denote stimuli where the right signal was quieter than the left signal. ILDs tested ranged from -16 to +18 dB depending on the frequency of the stimuli.

Stimuli were presented using cross-talk cancellation to eliminate undesired signal paths between the loudspeakers and their respective contralateral ears [29] (Fig 1B). We used the same cross-talk cancellation filters in all animals. They were based on head-related transfer functions (HRTFs) measured with probe microphones (ER-7C, Etymotic Research Inc.) in three gerbil carcasses, each measured with at least three slightly different head positions. A single set of measured HRTFs from two symmetric speakers (Fig 1A) was selected to serve as source for the cross-talk cancellation process.

The cross-talk cancellation filters were calculated with the following algorithm: First, the impulse responses were multiplied with a 4 ms raised cosine window in order to eliminate late room reflections and to smooth out subject-individual variations (which cannot be utilized by the other subjects during the experiments) in the spectrum of the HRTFs. From the windowed impulse responses, a (digital) Fourier transform was calculated. The complex values of corresponding spectral bins from all four speaker/ear combinations form a 2x2 matrix *H*(*ω*) (left to left and right to right on the diagonal, the cross terms on the remaining entries, where the first index corresponds to the sending speaker and the second one to the receiving ear):

$$H\left(\omega \right)=\left[\begin{array}{cc} H_{L,L}\left(\omega \right) & H_{R,L}\left(\omega \right)\\ H_{L,R}\left(\omega \right) & H_{R,R}\left(\omega \right) \end{array}\right]$$ (1)

Inverting each of these matrices yields the cross-talk cancellation filters *X*(*ω*):

$$X\left(\omega \right)=H^{-1}\left(\omega \right)=\frac{1}{H_{L,L}\left(\omega \right)H_{R,R}\left(\omega \right)-H_{R,L}\left(\omega \right)H_{L,R}\left(\omega \right)}\left[\begin{array}{cc} H_{L,L}\left(\omega \right) & -H_{L,R}\left(\omega \right)\\ -H_{R,L}\left(\omega \right) & H_{R,R}\left(\omega \right) \end{array}\right]$$ (2)

We verified that no ill-conditioned matrices (i.e., with undefined inverse) occurred. Frequency bins below 500 Hz and above 20 kHz were set to zero in order to avoid artifacts before re-transforming the resulting spectra into the time domain.

Stimuli were first generated for the left and right ear (their spectra denoted *L*(*ω*) and *R*(*ω*), respectively) with symmetrically distributed interaural disparities, filtered with the cross-talk cancellation filters, added up for each loudspeaker and then presented from the loudspeakers at -18 and +18° from the midline (Fig 1A) at a mean level of 60 dB SPL randomly roved within ±3 dB.

The choice of the combination of speaker pair and measured set of HRTFs was based on several factors: (1) Sound paths were least obstructed by essential parts of the experimental setup (e.g, light barriers or tubes delivering reward pellets). (2) Small head movements result in lower error of sound path length for small than for large speaker separations. (3) Post-processing the chosen set of cross-talk cancellation filters with different HRTFs than the source HRTF set (i.e., other head positions and/or subjects) resulted in the smallest deviation from the ideal impulse response among all combinations of mismatched source HRTFs and post-processing HRTFs.

$$\underset{\begin{array}{c} \text{ear}\\ \text{signals} \end{array}}{\underset{︸}{\left[\begin{array}{c} L_{\text{ear}}\left(\omega \right)\\ R_{\text{ear}}\left(\omega \right) \end{array}\right]}}=\underset{\begin{array}{c} \text{transfer}\\ \text{functions} \end{array}}{\underset{︸}{H\left(\omega \right)}}\cdot \underset{\begin{array}{c} \text{presented}\\ \text{signals} \end{array}}{\underset{︸}{X\left(\omega \right)\left[\begin{array}{c} L\left(\omega \right)\\ R\left(\omega \right) \end{array}\right]}}=\left[\begin{array}{cc} 1 & 0\\ 0 & 1 \end{array}\right]\cdot \left[\begin{array}{c} L\left(\omega \right)\\ R\left(\omega \right) \end{array}\right]$$ (3)

### Technical constraints of the virtual-headphones approach

The actual signals at the ears of subject carcasses that were different from the one used for the generation of the filters, and with slightly different head positions, were examined for artifacts. For the frequencies at which ITD discrimination was tested, unambiguous ILDs or ILDs exceeding threshold values (determined in a pilot study) were not present in the virtual-headphone stimuli resulting in the ITD as the only usable cue to perform in a left/right discrimination task (S1 Fig, top row). At the frequencies at which ILD discrimination was tested, unwanted ITDs were either below ITD threshold (determined in a pilot study) or ambiguous making them unreliable cues in a left/right discrimination task (S1 Fig, bottom row).

Cross-talk cancellation yields near-perfect results only for exactly the same subject and exactly the same position that was used for measuring the HRTFs on which the processing was based [31,32]. In our experiments, meeting these constraints was impossible: (1) We used gerbil carcasses to measure HRTFs, and (2) though the experimental gerbils kept their head still during a single trial, the head position they assumed varied between trials. The variation in head position, however, was small and centered positions dominated, i.e., extreme head positions occurred only infrequently. This was confirmed by analyzing video recordings of three subjects during free-field experimental sessions. The standard deviations of head azimuth ranged between 4° and 6°, and the standard deviations of head displacement ranged between 1.2 mm and 2.5 mm. To estimate the errors resulting from the occurrent head positions and the inter-subject variability, we processed the virtual-headphone stimuli with HRTFs measured from different individuals (i.e. carcasses) and at different head positions matching the ones observed during the experiments. The analysis yielded ITDs and ILDs that would be produced at the ears in the case of using non-individualized HRTFs in potentially misaligned head positions (S1 and S2 Figs). Due to the small range of animal movement during the experiments, we divided the reproduction errors into head-position dependent and head-position independent components. The latter depend on frequency and individual subject and only result in a shift of response bias (“accuracy”), while the former vary within an experimental session and may affect the measured thresholds (“precision”). The mean ITD reproduction error resulting from head movement of a single individual was between 5 and 9 μs for desired ITDs between -120 and +120 μs. The fixed component of the ITD reproduction error was below 1 μs for frequencies up to 1250 Hz and between 7 and 11μs for frequencies above 1250 Hz. The mean ILD reproduction error resulting from head movement of a single individual was approximately 1, 2, and 2 dB for 2, 4, and 6 kHz, respectively, for desired ILDs between -12 and 12 dB, and the fixed component was 1, 3, and 6 dB for 2, 4, and 6 kHz, respectively. Individual and position mismatch also produce unwanted artifact non-zero ILDs or ITDs along with desired ITDs and ILDs, respectively. The range of these artifacts is in the same order of magnitude as the errors mentioned above. In addition, they are strongly dependent on the actual head position and not consistently conflicting or non-conflicting with the desired cue, thus can be assumed to be invalid as a reliable alternative or additional cue.

### Procedure

Gerbils were trained in a left/right discrimination task using operant conditioning under positive reinforcement. During initial training, broadband noise bursts presented exclusively under free-field conditions from the four outermost loudspeakers (±50 and ±90°) were used. The animals learned to initiate the start of a trial by mounting the pedestal on the Y-shaped platform and poking their nose in the half-ring-shaped poke-hole (Fig 1A, inset), thus ensuring a reproducible head position. Animals were trained to remain in that position until the presentation of a sound after a random waiting interval of 500 to 2500 ms. On perceiving the sound, animals had to respond by approaching the feeding bowl either in the left or in the right arm of the Y-shaped platform. Correct responses triggered a food reward (Fig 1A). Food rewards were given with a probability of 80%. Incorrect responses remained unrewarded. Trials with an ILD of 0 dB or an ITD of 0 μs were rewarded with a probability of 50%. Trials with an ITD outside the range that gerbils can naturally experience (-130 to 130 μs, [13]) were also rewarded with a probability of 50% so as to not bias animals' responses. If the animal remained in the half-ring-shaped poke-hole >3 s after the onset of the sound or did not approach either feeding bowl within 5 s after leaving the half-ring-shaped poke-hole, the response was registered as a missed stimulus trial and excluded from the analysis. The experimental setup and procedure were controlled using custom software.

### Data collection

A single free-field session comprised 84 trials including four warm-up trials at the beginning. During warm-up trials, stimuli from loudspeaker positions at ±30 and ±50° were presented. Ten repetitions per loudspeaker position (n = 8) were tested. Free-field sessions were included in the analysis if they fulfilled the following criteria: (1) Animals responded with ≥80% correct to sounds emitted from the outermost loudspeakers (±50°). (2) Percentage correct responses for the right innermost loudspeakers (+6°, +12°) were within 30% of the percentage correct responses for the left innermost loudspeakers (-6°, -12°). The first session of a stimulus condition was excluded from analysis (irrespective of being valid or invalid). Then the animals typically needed a total of four sessions (median: 4, quartiles: 3 and 6, 10^th^/90^th^ percentiles: 3 and 8) in order to complete three valid session. Three valid sessions per stimulus type per animal were merged for the construction of psychometric functions. Because missed stimulus trials were excluded from the analysis (no response to the right or to the left could be determined on such trials), the merging of sessions ensured that 25 to 30 trials per loudspeaker position were analyzed (median: 30, quartiles: 29 and 30, 10^th^/90^th^ percentiles: 28 and 30 trials). In the frequency condition 1250 Hz, two out of six animals collected four valid sessions and two out of six animals collected five valid sessions resulting in a minimum of 35 (out of 40) trials and 49 (out of 50) trials being analyzed in those animals. The number of missed trials was independent of loudspeaker position and frequency condition. Missed stimulus trials occurred in about 2% of all trials presented.

A single virtual-headphone session comprised 74 trials. In the four warm-up trials at the beginning of a session, the two largest absolute ILD or ITD values tested in a session were presented. Ten repetitions per ILD or ITD (n = 7) were tested in a single session. In the experiments testing responses to ITD values outside the naturally occurring range of the gerbil, the number of ITD tested was greater and the total number of trials per session deviated correspondingly. The ITD values that were then used in the warm-up trials were the largest absolute ITD values within the naturally occurring range (<130 μs, [13]). All sessions but the first one were included in the analysis, i.e. usually three sessions, resulting in 30 stimulus presentations per ITD or ILD. Due to missed stimulus trials, a minimum of 25 trials per ITD or ILD per animal were analysed (median: 30, quartiles: 29 and 30 trials). Missed stimulus trials across all conditions and animals made up ~1% of the total number of trials; their probability of occurrence was independent of the magnitude of the ILD or ITD applied. In the experiments testing ITD sensitivity of frequencies ≥2000 Hz, sessions often comprised only 39 trials (including four warm-up trials at the beginning), i.e., five repetitions per ITD were presented during a single session. We excluded sessions where the data was non-monotonic, and animals were run until at least a minimum of 20 trials per ITD were presented. Up to 40 trials per ITD were presented to some animals in the different frequency conditions. Hence, the animals' psychometric functions for testing the ITD sensitivity of frequencies ≥2000 Hz were based on 19 to 40 trials per ITD (median: 30, quartiles: 28 and 35 trials). Missed stimulus trials again occurred in about 1% of all trials presented.

Animals were tested in blocks with the stimulus type kept constant throughout a block. The order, in which animals were tested for the different stimulus types, was randomized resulting in the blocks that tested the free-field performance and the performance under virtual-headphone conditions to be interleaved.

### Data analysis

#### Psychometric functions

Psychometric functions were created by expressing performance as 'percentage response to the right'. Virtual-headphone stimulation using ILD or ITD each resulted in one psychometric function per stimulus type. Free-field stimulation yielded two psychometric functions for each animal and stimulus type: (1) performance vs. free-field ILD and (2) performance vs. free-field ITD (Fig 1C). The free-field ILD and free-field ITD values at the eight angular positions were derived from fits of cubic polynomials to ILD and ITD values that were averaged for each angular position from the directional transfer functions of three gerbil carcasses in up to four positions (S3 Fig). That means we used the same ILD and ITD values in all animals tested in the present study.

#### Thresholds

Cumulative normal distribution functions with four parameters (slope, inflection point, offset from 0, and offset from 1) were fitted to the psychometric functions (Fig 1C, Eq 4):

$$\left(x\right)=\alpha +\left(1-\alpha -\beta \right)\Phi \left(\frac{x-\mu }{\sigma }\right)$$ (4)

where *p*(*x*) is the probability of the answer “right”, *x* denotes the respective parameter, ILD or ITD, *Φ*(∙) is the cumulative normal distribution function, and *σ*, *μ*, *α*, and *β* are slope, inflection point, offset from 0, and offset from 1, respectively.

Thresholds were calculated as the difference between the ITD or ILD at the inflection point and at the point 0.26 above the inflection point (Fig 1C), thus corresponding to a d'-value of 1 [33] and being shown to not be influenced by spatial response biases [30]. Calculating thresholds that way assumed that subjects discriminate the position of the sound source on the interaural axis (i.e. within the head) relative to an internal representation of the midline. In the case of free-field sound localization, it assumed that subjects use a non-acoustic reference angle (e.g. 0° azimuth) to discriminate the angular location of the sound [30]. Thresholds were included in the analysis and the calculation of average threshold values, if the cumulative normal distribution functions from which they were derived reached a coefficient of determination R^2^≥0.875 when fit to the raw data. This value was chosen so as to maximize the number of thresholds used in subsequent analyses while removing data sets which contained too much noise to be analyzed adequately. We assume that the subjects’ responses are biased by both an individual perception of the center between left and right stimuli, and a general individual asymmetric preference of left/right responses independent of the perceived stimulus location. Both biases are reflected in the psychometric function: the former in a shift of the psychometric function along the ITD or ILD axis, and the latter in a deviation of the inflection point from the 0.5 function value resulting from differing offsets from 0 and 1.

#### Statistical testing

Statistical tests of significance were conducted on thresholds using a generalized linear mixed model analysis of variance (GLMM ANOVA, IBM SPSS Statistics, Version 24.0). *Post hoc* analyses were performed using the Bonferroni correction to correct for multiple comparisons. Some comparisons could not be performed because of the small sample size and thereby reduced statistical power.

## Results

### Sensitivity to ITD

**Fig 2:**
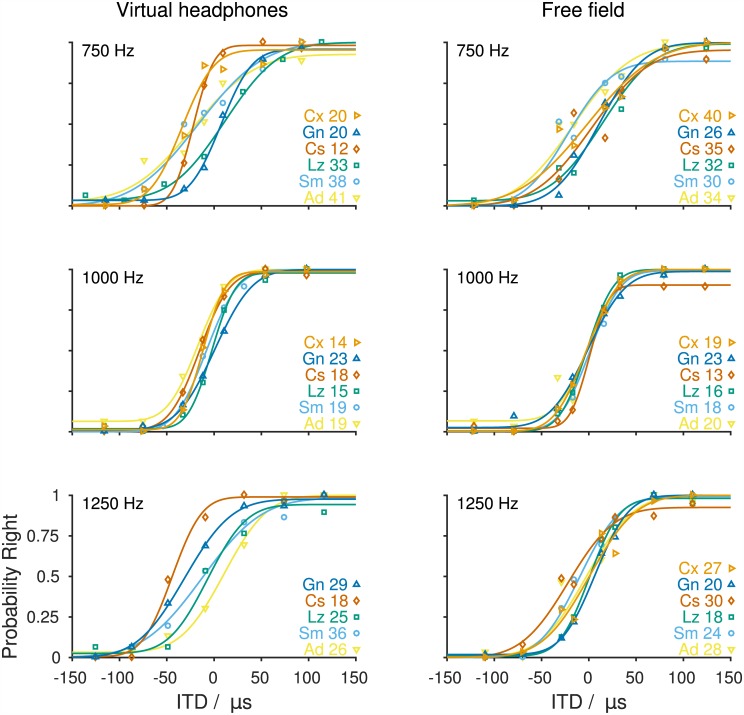
Probability of responses to the right as a function of ITD at 750, 1000, and 1250 Hz. The left column shows the performance using virtual headphones. The right column shows the free-field performance depending on the ITDs occurring in the free-field experiments. Symbols indicate individual performance of animals [750 Hz: n = 6 (vhp, free-field), 1000 Hz: n = 6 (vhp, free-field), 1250 Hz: n = 5 (vhp) and 6 (free-field)]. Lines represent the cumulative normal distribution functions with four parameters (slope, inflection point, offset from 0, offset from 1) fitted to the raw data of individual animals. Identical colors and symbols represent data and fit of the same individual animal in the different panels. Thresholds (in μs) and identifiers of individual animals are given on the right.

**Fig 3:**
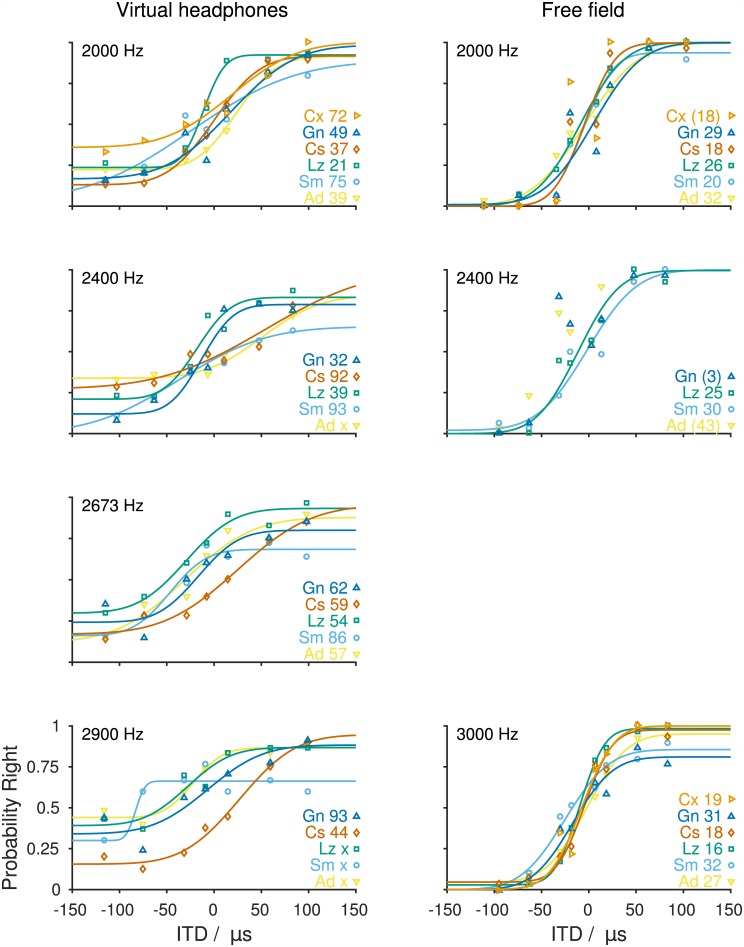
Probability of responses to the right as a function of ITD at frequencies ≥2000 Hz. The left column shows the performance using virtual headphones. The right column shows the free-field performance depending on the ITDs occurring in the free-field experiments. Tones with frequencies of 2000 [n = 6 (vhp, free-field)], 2400 [n = 5 (vhp) and 4 (free-field)], 2673 (n = 5, vhp only), 2900 Hz (n = 5, vhp only), and 3000 Hz (n = 6, free-field only) were presented. Symbols and lines as in the previous figure. Thresholds (in μs) and identifiers of individual animals are given on the right. ‘x’ indicates that no threshold could be determined because the threshold criterion was not reached. Thresholds in brackets were derived from cumulative normal distribution functions that yielded R^2^<0.875 with the raw data and not used in further analyses; such cumulative normal distribution functions are not shown.

ITDs are thought to be the dominant sound localization cue at low frequencies [34,35]. Gerbils' sensitivity to ITDs was therefore measured for frequencies between 750 Hz and 2.9 kHz. Gerbils' responses to ITD depended on the frequency that an ITD was applied to (Figs 2 and 3). At frequencies <2 kHz, gerbils' psychometric functions had steep slopes and only minor offsets from 0 and 1, i.e., the gerbils consistently responded to the left side when tones with large negative ITDs were presented and to the right for large positive ITDs (Fig 2). The coefficients of determination yielded between the cumulative normal distributions and the raw data were between 0.961 and 0.999 (median: 0.996, quartiles: 0.990 and 0.999; S4 Fig). Also, performance between animals showed only moderate amounts of variability, with the most consistent performance across animals found at a frequency of 1 kHz. At frequencies ≥2 kHz, the pattern changed (Fig 3). The gerbils still approached the left side for large negative ITDs and the right side for large positive ITDs but performance often did not reach probabilities of 0 and 1. Additionally, psychometric functions at frequencies from 2 to 2.9 kHz had shallower slopes and smaller coefficients of determination (0.853 to 0.996, median: 0.940, quartiles: 0.920 and 0.974, S4 Fig), all indicating a reduced sensitivity to ITD at those frequencies. Another indicator of poorer ITD sensitivity was the number of sessions that the animals had to complete to allow merging of at least three sessions where the probability of response to the right increased monotonically with ITD. While at frequencies <2 kHz data from three out of three data collection sessions could be merged, animals completed between three and eight data collection sessions at frequencies ≥2 kHz to gather data from three data collection sessions showing a monotonic increase of the probability of responses to the right with ITD.

**Fig 4:**
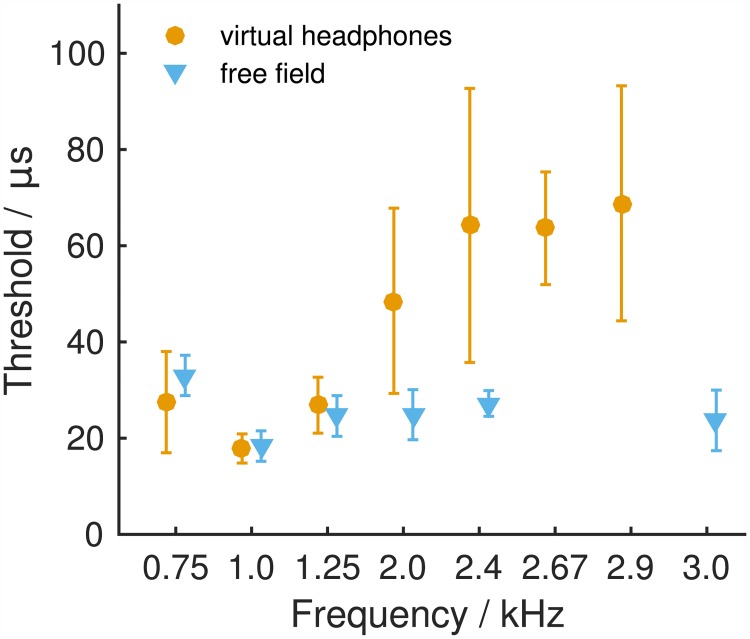
Mean ITD thresholds measured under virtual-headphone stimulation (vhp, orange) and derived from free-field stimulation (ff, blue). Only threshold values calculated from cumulative normal distribution functions that yielded an R^2^>0.875 with the raw data were included in the calculation of the average threshold values (S3 and S4 Tables, Figs 2 and 3). Error bars show standard deviations.

Individual ITD thresholds across frequencies varied between 12 and 93 μs (S3 Table). Mean ITD thresholds were 27, 18, 27, 49, 64, 64, and 69 μs for 750, 1000, 1250, 2000, 2400, 2674, and 2900 Hz, respectively (Fig 4, orange). Thresholds could not be determined for one out of the five animals tested at a frequency of 2400 Hz and for three out of the five animals tested at a frequency of 2900 Hz because psychometric functions did not reach threshold criteria. At frequencies >2900 Hz, ILD cues, due to the constraints of the virtual headphones, became too large to exclude the possibility that the gerbils used those instead of the applied ITD to solve the left/right task. We therefore refrained from testing gerbils' ITD sensitivity at higher frequencies. A GLMM ANOVA with fixed factor frequency (0.75, 1.0, 1.25, 2.0, 2.4, 2.67, and 2.9 kHz) and subject included as random factor detected a highly significant influence of frequency on ITD thresholds (F(6,27) = 6.290, *p<0*.*001*). *Post hoc* analyses showed significant differences between the frequencies from 0.75 to 1.25 kHz with the frequencies from 2.4 to 2.9 kHz (*0*.*001≤p≤0*.*036*).

To infer the use of ITD information during sound localization in the free field, ITD thresholds were derived from the animals’ free-field performance. Angular positions were transformed to ITD using the ITD values occurring under the free-field stimulation (Fig 1C, S3 Fig). In contrast to ITD thresholds measured under virtual-headphones conditions, ITD thresholds derived from free-field stimulation did not show a strong dependence on frequency. Individual free-field ITD thresholds lay between 13 and 40 μs (S4 Table) with mean thresholds of 33, 18, 25, 25, 27, and 24 μs for 750, 1000, 1250, 2000, 2400, and 3000 Hz, respectively (Fig 4, blue). A GLMM ANOVA with the fixed factor frequency (0.75, 1.0, 1.25, 2.0, 2.4, and 3.0 kHz) and subject included as random factor detected a significant influence of frequency on free-field ITD thresholds (F(5,26) = 5.379, *p = 0*.*011*). *Post hoc* analyses showed a significant difference of thresholds measured at 750 Hz and 1 kHz (*p = 0*.*001*).

While at frequencies ≤2.0 kHz ITD sensitivities measured under virtual-headphones condition and free-field stimulation appear to be very similar, they clearly deviate from each other at frequencies >2.0 kHz (Fig 4). A GLMM ANOVA with fixed factors frequency (0.75, 1.0, 1.25, 2.0, 2.4, and 2.9/3.0 kHz) and mode of acquisition (virtual-headphones and free-field) and subject included as a random factor showed a highly significant influence of mode of acquisition (F(1,48.104 = 19.975, *p<0*.*001*) and of frequency (F(6,47.282) = 8.078, *p<0*.*001*) on ITD thresholds. Further, there was a significant interaction of the two fixed factors (F(5,47.124) = 5.382, *p = 0*.*001*). *A post hoc* analysis could only be conducted at frequencies ≤2 kHz due to the small sample size and therefore the lack of statistical power at higher frequencies (2.4 kHz free-field: n = 2, 2.9/3.0 kHz virtual-headphones: n = 2). Virtual-headphone ITD thresholds and free-field ITD thresholds were not significantly different for frequencies ≤2 kHz (*0*.*132≤p≤0*.*727*).

### Responses to ITDs beyond the naturally occurring ITD range

**Fig 5:**
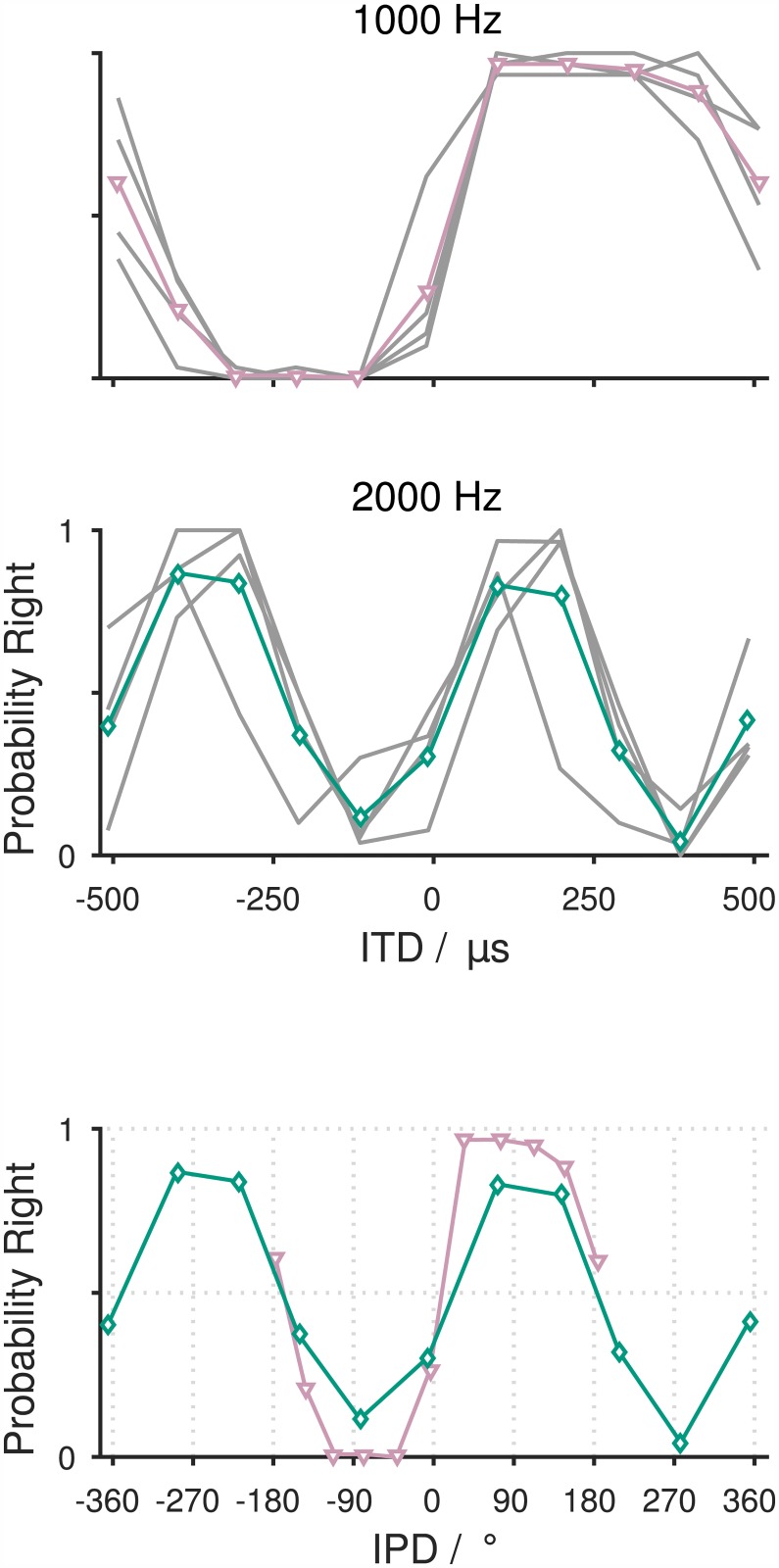
Behavioral responses to tones with ITD outside the naturally occurring range. **(A)** Probability of responses to the right as a function of ITD measured at 1000 and 2000 Hz for an ITD range between -510 and 510 μs. Responses of four animals are shown in grey. The mean responses at 1000 and 2000 Hz are depicted in purple (triangles) and green (diamonds), respectively. **(B)** Probability of response to the right as a function of IPD measured at 1000 Hz (purple) and 2000 Hz (green).

The virtual-headphone stimulation allows the presentation of stimuli that, under natural listening conditions (i.e. under free-field stimulation), cannot be experienced by an individual. Four gerbils were tested with tones with a frequency of 1000 or 2000 Hz with the ITD ranging between -510 and 510 μs. In the gerbil, naturally occurring ITDs lie between -130 and 130 μs [13], i.e., most of the ITDs tested in this experiment were outside the ITD range gerbils naturally experience. Gerbils' left/right responses to these stimuli changed periodically (Fig 5A, S5 Table). The period length of those changes matched the period length of the stimulation frequency. In the case of 1000 Hz, responses ran through a full periodic cycle within the approximately 1000 μs range of ITD tested (top of Fig 5A). In the case of 2000 Hz, responses showed two full cycles within the approximately 1000 μs range of ITD tested (bottom of Fig 5A). The cyclic pattern can be compared between stimulation frequencies when ITDs are transformed to interaural phase delays (IPDs) (Fig 5B). The probability of animals’ responses to the right monotonically increased from -90° to +90°. In this IPD range, one of the two sound waves that reach either ear clearly leads in time, matching the sign of the ITD. Around ±270°, the probability of responses to the right was opposite from what would have been expected from the sign of the corresponding ITDs. At an IPD of -270° the corresponding ITD is negative but the probability of responses to the right was high, while at an IPD of 270°, corresponding to a positive ITD, the probability of responses to the right was low. At IPD ±360°, 0°, and ±180°, the probability of responses to the right was about 0.5. In a left/right discrimination task, this can be explained by the absence of delays between the sound waves reaching the two ears with an IPD of 0° and ±360° and the impossibility to assign a leading wave and a lagging wave to the two sound waves reaching the ears with an IPD of ±180°.

### Sensitivity to ILD

**Fig 6:**
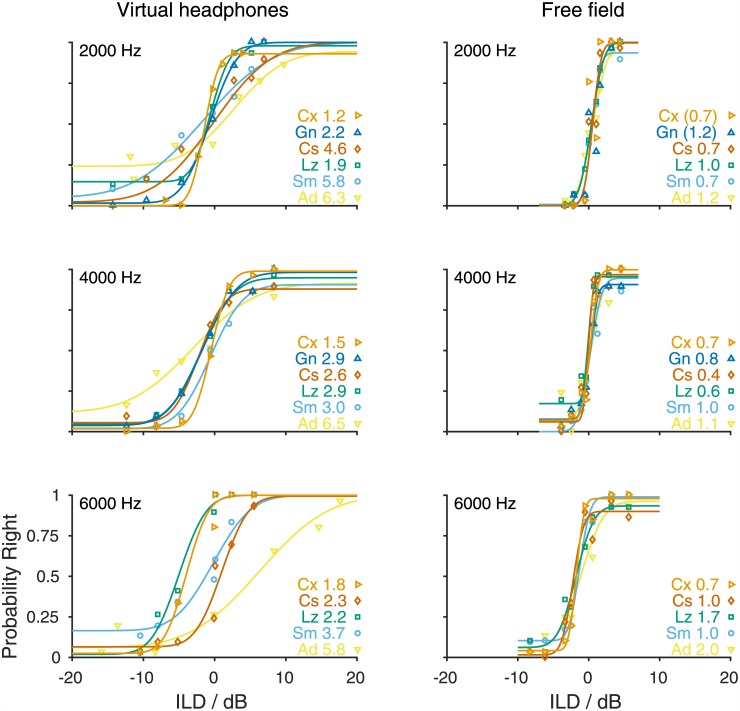
Probability of responses to the right as a function of ILD obtained with tones of 2000, 4000, and 6000 Hz. Symbols show individual performance of animals (2000 Hz: n = 6, 4000 Hz: n = 6, 6000 Hz: n = 5). Lines represent the cumulative normal distribution functions fitted to the raw data of individual animals. Identical colors and symbols represent data and fit of the same individual animal in the different panels. Thresholds (in dB) and identifiers of individual animals are given on the right.

**Fig 7:**
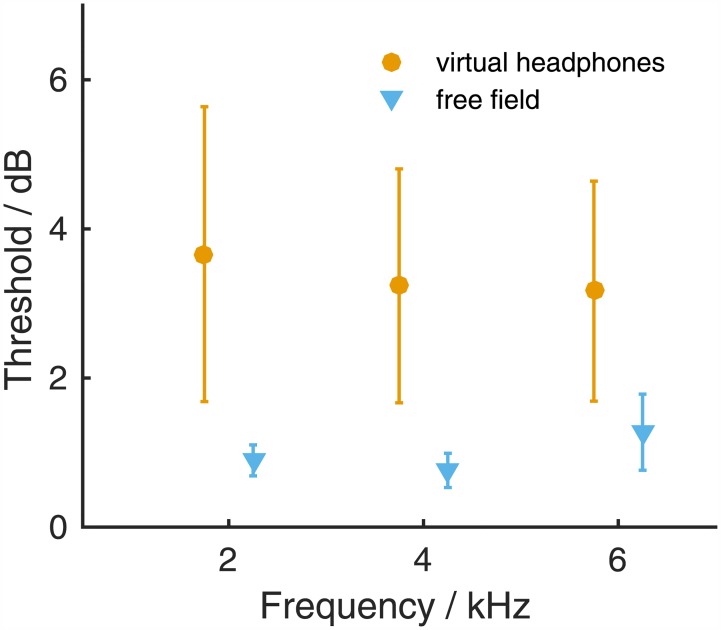
Mean ILD thresholds measured under virtual-headphone stimulation (vhp, orange) or derived from free-field stimulation (ff, blue). Thresholds calculated from cumulative normal distribution functions that reached R^2^>0.875 with the raw data were included in the calculation of average thresholds. Error bars show standard deviations.

Next to ITDs, ILDs are the other important sound localization cue used to localize sounds in the horizontal plane. We therefore measured gerbils' ILD sensitivity for the frequencies 2, 4 and 6 kHz, i.e. at frequencies for which the presentation of signals with an ILD with virtual headphones was possible. All gerbils were able to lateralize tones with an ILD (Fig 6). ILD thresholds ranged between 1.2 and 6.5 dB (Fig 6, left column; S6 Table). Mean ILD thresholds were 3.7, 3.2 and 3.2 dB for 2, 4 and 6 kHz, respectively (Fig 7, orange). ILD thresholds derived from the free-field performance were much smaller: They ranged from 0.4 to 2.0 dB (Fig 6, right column; S7 Table), and mean free-field ILD thresholds were 0.9, 0.8 and 1.3 dB for 2, 4 and 6 kHz, respectively (Fig 7, blue). A GLMM ANOVA with fixed factors frequency (2, 4 and 6 kHz) and mode of acquisition (virtual-headphones and free-field) and subject included as a random factor did not detect a significant influence of frequency on ILD thresholds (F(2,21.491) = 0.051, *p = 0*.*951*) but showed a highly significant difference between ILD thresholds obtained using virtual-headphones stimulation and ILD thresholds derived from free-field performance (F(1,21.288) = 45.052, *p<0*.*001*). There was no significant interaction of the two fixed factors (F(2,21.279) = 0.843, *p = 0*.*444*).

## Discussion

### Virtual-headphone ITD sensitivity

The ITD sensitivity that we observed in the gerbil very well matched the ITD sensitivities reported from other species. It was frequency-dependent with the highest sensitivity observed at 1 kHz and steadily decreasing with increasing frequency. The highest sensitivity at 1 kHz or a frequency region that encompassed 1 kHz has also been observed in humans [14,17,36] and ferrets [22] and is very likely the region of highest ITD sensitivity in monkeys [21] and cats [27]. In all those species, ITD sensitivity declines below and/or above the frequency region around 1 kHz (if tested). Considering how well data on the ITD sensitivity of the gerbil resemble the data on ITD sensitivity in other species, the approach of using virtual headphones seems a suitable tool to investigate questions of binaural hearing with a focus on low-frequency ITD information.

The increase in ITD thresholds with increasing frequency is thought to stem from the poorer preservation of temporal information contained in the stimulus, in the form of phase locked responses of neurons, as the stimulus frequency increases [14,37]. In the gerbil, strongest phase locked responses of auditory nerve fibers can be found at 500 Hz; the strength of phase locked responses steadily decreases from around 1 kHz to 4 to 5 kHz [38]. This decline could explain the rise in ITD thresholds with increasing stimulus frequency in the present study. In the ferret, the upper limit of phase locked responses in auditory nerve fibers [39] coincides with the upper limit of ITD sensitivity [22]. It seems likely that this might also be the case in the gerbil, particularly when considering that the gerbil's small head size renders ITD information, even at those high frequencies, unambiguous, thus, providing useful binaural information at such high frequencies [14,22]. Limited by the constraints of the virtual-headphone approach, however, we were not able to test the upper frequency limit of behavioral ITD sensitivity but we still argue in the section where we compare virtual-headphone and free-field sensitivities that ITD sensitivity at frequencies >2.9 kHz may contribute to sound localization.

### Lateralization of phase-ambiguous signals

When we presented tones with ITDs outside the naturally occurring range of the gerbil [13], the probability of right responses changed in a periodic way. That is to be expected if the animals used phase information to lateralize the tones presented. As long as the ITD applied to the tones corresponds to an IPD value well within ±90°, lateralization will be in the direction of the side of the ear at which the sound wave leading by ITD arrives [40,41]. The gerbils very likely perceived a clearly lateralized signal to the left or to the right as suggested by studies in owls and humans [25,40,41]. Notably, the range for which the gerbils most likely perceived a clearly lateralized percept extended to absolute ITDs of up to ~300 μs for tones with a frequency of 1000 Hz as indicated by the probability of right responses near or at 0 and 1 at absolute ITDs between about 100 and 300 μs. Considering that reflections from the ground and obstacles can substantially enlarge the naturally occurring ITD range [42], this might seem less surprising. Also, there are neurons in the medial superior olive, i.e. the first neurons in the ascending auditory pathway that receive binaural information, that show their maximum firing rates in response to signals with ITDs that lie well outside the naturally occurring range [1,12,43,44]. Such neurons might form the neural basis for the consistent left or right responses of the gerbils to tones with an absoulte ITD of up to ~300 μs. At an ITD corresponding to an IPD of ±180°, gerbils approached the right loudspeaker with a probability of about 0.5. Humans perceive an ambiguous percept for tones with such an IPD, i.e., two sounds may be heard, one on each side, and perceived lateral positions were reported to be on the left and the right side of the head [40,41]. Perceiving two sounds or a sound that is not well lateralized to either side might thus explain the 0.5 response probability to the right that we observed in the gerbils. At an ITD corresponding to an IPD close to ±270°, gerbils consistently chose to respond to the side that lagged by ITD suggesting that the perceived sound had a clear lateral position. The perceived lateral position corresponded to the smaller of the two possible IPD/ITD values between the sound wave reaching the left ear and the sound wave reaching the right ear [45].

### Virtual-headphone ILD sensitivity

ILD sensitivity varies between species, much more so than ITD sensitivity. ILD sensitivity can be as small as 0.5–1.2 dB measured in humans [18,46] and cats [27] but can reach higher values, depending on frequency. Average maximum values found for example in ferrets were ~3 dB [22], ~7 dB in pig-tailed macaques [21] and 3–5 dB in aged humans [15]. Anatomical characteristics are probably decisive for the species-specific variability. A large head, as in humans, is advantageous because it leads to a strong head shadowing effect. The same is likely true for movable pinnae, as observed in the cat [47]. A high ILD sensitivity of a species might therefore stem from the availability and thus the behavioral relevance of ILD cues. A small head induces sizable ILDs only at higher frequencies (see [13] for the gerbil) at which also spectral sound localization cues become available. In the gerbil, ILD cues might therefore function as complementary rather than primary cues for azimuthal sound localization, in the mid-frequency range complementing ITD cues and in the high-frequency range complementing spectral cues. Strikingly, the ILD sensitivity varied greatly between individuals, particularly when contrasted with the free-field ILD sensitivity. The smallest ILD thresholds were <2 dB, while the largest ILD thresholds were ~6 dB. This may indicate that individual gerbils weighed ILD and ITD cues differently. ILDs might therefore serve as potent cues for some but not for other individuals.

### Comparison of virtual-headphone and free-field binaural sensitivities: Implications for the use of binaural cues in the free field

Because we tested the same individual animals both under virtual-headphone stimulation and free-field stimulation, we were able to directly compare performance under both listening conditions. We infer the contribution of ITD and ILD to sound localization in the free field at certain frequencies by assuming that either exclusively ITD or exclusively ILD were used when localizing sounds of a particular frequency (Fig 1C). Virtual-headphone and free-field thresholds that match in magnitude suggest that the cue tested is sufficient to explain localization performance in the free field. Virtual-headphone ITD and ILD sensitivities larger than free-field ITD and ILD sensitivities imply the use of additional cues when localizing sounds in the free field.

### Free-field thresholds pose a valid comparison for virtual-headphone thresholds

Before we can interpret the virtual-headphone thresholds in light of the free-field thresholds, we need to verify that the free-field performance we measured here was representative for the gerbil’s free-field sound localization performance and could thus serve as the basis for a valid comparison with the virtual-headphone performance. Four earlier studies investigated gerbils’ sound localization of tones or narrow-band signals between 500 Hz and 20 kHz [9–11,48]. To compare data across studies, we calculated or extracted minimal resolvable angles (MRAs, for details, see [11]) from the present data and data from three of the four previous studies (S5 Fig). In general, the MRAs across studies and frequencies fell within the same range. Exceptions were the MRAs at 1 kHz, 2.4 kHz and at frequencies >4 kHz. Free-field sound localization of 2.4 kHz tones has not been tested before. The current MRAs were higher than what would have been expected from linear interpolation of previously collected data [11]. This might be indicative of a frequency range for which poor sound localization is observed because neither ITD nor ILD cues can fully be exploited. This has also been suggested before for 2.8 kHz [9]. At 1 kHz, the MRAs measured in the present study and in a study using the same setup but different individuals trained in a slightly different task ([48] measured absolute and relative sound localizations in the same sessions) were much lower than the MRAs measured previously [11]. At frequencies >4 kHz, the MRAs measured in [11] also tended to be higher than the ones obtained in the present study and in [48]. Very likely the testing of the individuals over extended periods of time in the left/right discrimination task for the present and the previous study by Tolnai and colleagues [48] led to those small MRAs. Those free-field performances can therefore be considered best possible free-field performance of highly trained gerbils. Because the same extensive exposure to the stimuli is true for the virtual-headphone stimulation, we conclude that the free-field data can serve as valid comparison for the virtual-headphone data.

#### Validity of the virtual-headphone stimulation

Having established that the free-field thresholds can duly be compared to the virtual-headphone thresholds, we now need to clarify whether inaccuracies in cue reproduction due to the stimulation via virtual headphones affect thresholds. We derived thresholds from simulated experiments in which the actual ILD (or ITD) was jittered with a normal distribution based on the measured reproduction errors (S1 Fig). The variance of this distribution was either based only on realistic variation in head position while keeping the error due to non-individual HRTFs fixed (“precision/head position” simulation), or on the total error including the component that was assumed to be fixed within an experimental session (“accuracy/worst case” simulation). The simulated responses were drawn from the psychometric function that was based on the mean free-field ILD (or ITD) threshold at a certain frequency and that connected free-field ILD (or ITD) with probability ‘right’ response. Twenty simulated responses per nominal ILD (or ITD) value yielded the psychometric function from which then an ILD (or ITD) threshold was derived. This was repeated 100 times resulting in a mean simulated threshold. We assume that the gerbils cannot have lower thresholds in the virtual-headphone experiments than those simulated ones because reproduction errors do not occur during free-field stimulation and head positioning during free-field and virtual-headphone experiments would have been identical. The head positions we assumed for the simulations were taken from a set of measured HRTFs that included head azimuths slightly larger than what we measured (see Methods). This corresponds to a variation of more than 6°. The simulated ILD and ITD thresholds therefore represent worst-case scenarios for thresholds due to inaccuracies in cue reproduction. The ILD thresholds in the “precision” simulation were less or equal to 1.3 (SD 0.2), 1.5 (SD 0.2) and 2.0 (SD 0.3) dB for 2, 4 and 6 kHz, respectively. The “accuracy” simulation, however, resulted in ILD thresholds of 1.4 (SD 0.2), 2.6 (SD 1.0), and 4.2 (SD 1.1) dB for 2, 4, and 6 kHz, respectively (S6 Fig). Simulated ITD thresholds of both types (“precision/head position” and “accuracy/worst case”) were 23 to 36 (all SD 3) μs and thus only slightly larger than the ITD thresholds derived from the gerbils’ free-field performance (S7 Fig).

To conclude, the impact that particularly the use of non-individualized HRTFs (next to the differences in head position between trials) has on the accuracy with which ILD stimuli can be presented can explain the discrepancy between virtual-headphone and free-field ILD thresholds at 4 and 6 kHz. The match between simulated ITD thresholds and virtual-headphone ITD thresholds measured at 750 to 1250 Hz suggests that virtual-headphone ITD thresholds indeed demonstrate the gerbil’s ITD sensitivity. Other factors than the ones that can be explained by the limitations of the virtual-headphone technique affected virtual-headphone ITD thresholds at ≥2 kHz and ILD thresholds at 2 kHz. The lack of naturally occurring cues in the virtual-headphone stimulation, which are used for sound localization at certain frequencies, and also the presence of undesired ITD and ILD cues might explain the gerbils’ virtual-headphone performance at frequencies between 2 and 4 kHz. The former will be discussed in the next section. The latter seems unlikely because undesired ITDs and ILDs were either smaller than ITD and ILD thresholds or ambiguous about the lateralization of a tone (S1 Fig, right column). Still, ambiguous ITDs and ILDs might have led to misguided responses and thus to shallower psychometric functions and larger thresholds compared to the performance in the free field where such conflicting cues were absent.

#### The use of binaural information at different frequencies

For tones with frequencies of 750 to 1250 Hz, ITD thresholds measured under virtual-headphone stimulation agreed with ITD thresholds derived from free-field performance. This suggests that gerbils localize sounds in this frequency range using primarily ongoing ITD information. This agrees with findings in humans that demonstrated that there was no benefit from onset/offset ITD cues at low frequencies in addition to ongoing ITD (200 and 1000 Hz tested by [41]). It is also in line with the absence of usable ILD cues at those frequencies in our acoustic measurements (S1 Fig, top right). ILD cues were either non-informative about the side of the stimulation or too small to be used leaving ongoing ITD the sole potent localization cue in the horizontal plane. At 2 to 2.9 kHz, the exclusive use of ongoing ITD information cannot sufficiently explain gerbils' free-field sound localization acuity. Also the limitations of the virtual-headphone technique cannot explain the larger virtual-headphone thresholds. The use of additional cues must therefore gain importance at those frequencies. Such additional cues for localization in the free-field could be onset and offset ITD cues. Humans were shown to use them when lateralizing high frequency tones [37]. While they are prominent cues under free-field listening conditions, we made sure that they were not available in the virtual-headphone stimulation. The most likely additional cue, however, is ILD. Gerbils were sensitive to ILD at 2 kHz. Those thresholds were larger than those determined from the gerbils’ free-field performance and larger than the simulated thresholds that considered the limitations of the virtual-headphone stimulation suggesting that ILD information complements ITD information as the primary localization cue at ≥2 kHz.

## Conclusions

The present study used cross-talk cancellation techniques to present tones with an ITD or an ILD to Mongolian gerbils. ITD thresholds were frequency-dependent, with the smallest thresholds found at 1 kHz. Comparison of ITD thresholds with ITD thresholds that were determined from gerbils' free-field performance indicated that ongoing ITDs are the main cue used for sound localization at <2 kHz. The periodic response patterns observed for tones with ITDs outside the naturally occurring range suggest that gerbils use phase information when lateralizing narrow-band signals. ILD thresholds showed no frequency-dependence in the narrow frequency range tested here. At 4 and 6 kHz, the differences between virtual-headphone ILD thresholds and ILD thresholds assumed from the gerbils' free-field performance could be well explained by limitations of the cross-talk cancellation technique. At 2 kHz, where the ILD cues can be reproduced more faithfully, the differences suggest the complementary use of ILD and ITD for sound localization.

## Supporting information

S1 Fig Actual binaural cues being presented depending on the nominal cues. **The upper row** shows actual ITD (left), IPD (middle), and ILD (right) when presenting tones of different frequencies (0.75, 1, 1.25, 2, 2.4, 2.67, 2.9 kHz, lines) in relation to the nominal ITD (left) or the nominal ITD expressed as IPD (middle and right). **The bottom row** shows actual ILD (left), IPD (middle), and ITD (right) when presenting tones of different frequencies (2, 4, and 6 kHz, lines) in relation to the nominal ILD. Symbols and error bars indicate mean and standard deviation. Dashed lines indicate perfect cue reproduction. HRTFs between left and right speakers with both left and right ears, respectively, were measured with Etymotic ER-7C probe microphones from two gerbil carcasses in up to seven positions. The HRTFs were subsequently used as transfer function matrix *H*(*ω*) in Eq 3 with the original cross-talk cancellation matrix *X*(*ω*) (i.e., the same as in the experiments) to calculate the actual signals at the ears and to derive actual ILDs and ITDs with respect to the nominal binaural cues of the input signals in the simulations.
(PDF)

S2 Fig Actual binaural cues being presented depending on the stimulation frequency. **The upper row** shows actual ITD (left), IPD (middle), and ILD (right) when tones of different frequency with an ITD were presented. **The bottom row** shows the actual ILD (left), IPD (middle), and ITD (right) when tones of different frequency with an ILD were presented. Symbols and error bars indicate mean and standard deviation. Dashed lines indicate perfect cue reproduction. For measurement and calculation details see S1 Fig.
(PDF)

S3 Fig Binaural cues during free-field stimulation. ITD (two top rows) and ILD (two bottom rows), measured for frequencies between 750 Hz and 6 kHz, change with the azimuthal position. ITD decrease slightly with increasing frequency, while ILD increase with increasing frequency. Symbols show the individual measurements from gerbil carcasses. The lines show the cubic polynomial fit used to calculate the ITDs and ILD for behavioral free-field measurements.
(PDF)

S4 Fig Coefficients of determination. The left panel shows the coefficients of determination R^2^ in the virtual-headphone experiments. Each symbol marks the R^2^ for the fitted cumulative normal distribution function relating a single animal’s probability right response to ITD or ILD each experimental condition. The right panel shows R^2^ values for the free-field experiments. The black crosses indicate R^2^ values when the angular position was transformed to ILD and the grey crosses indicate R^2^ values when the angular position was transformed to ITD. The dashed lines indicates the criterion R^2^ = 0.875. Only threshold values derived from functions with R^2^>0.875 were included in the calculation of the average threshold for a certain experimental condition.
(EPS)

S5 Fig Free-field performance across studies and frequencies. Minimal resolvable angles (MRAs) were calculated as in [11] (golden squares) for the current data set (magenta crosses) and the data from [10] (red triangles) and [48] (green diamonds). The MRA corresponds to the speaker separation at the 75% correct response value of a psychometric function that is constructed by averaging correct responses of left and right stimulus presentations and plotting average correct responses versus speaker separation (for details, see [11]).
(EPS)

S6 Fig Simulation of ILD thresholds. We derived ILD thresholds from simulated virtual-headphone experiments to clarify the impact of inaccuracies in cue reproduction due to errors in cue precision (red squares) and cue precision plus accuracy (green diamonds) on ILD thresholds. Measured virtual-headphone ILD thresholds (orange circles) and free-field ILD thresholds (blue triangles) are shown for comparison. See main text for details. Symbols and error bars indicate medians and quartiles, respectively.
(EPS)

S7 Fig Simulation of ITD thresholds. We derived ITD thresholds from simulated virtual-headphone experiments to clarify the impact of inaccuracies in cue reproduction due to cue precision (red squares) and cue precision plus accuracy (green diamonds) on ITD thresholds. Measured virtual-headphone ITD thresholds (orange circles) and free-field ITD thresholds (blue triangles) are shown for comparison. See main text for details. Symbols and error bars indicate medians and quartiles, respectively.
(EPS)

S1 Table Data tables accompanying S1 and S2 Figs. Two gerbil carcasses (A, B) were measured in five (gerbil B) and seven different head positions (gerbil A) representing a combination of head orientation (central, left, right) and the additional deviation from the central position (back, front, far). The worksheets correspond to the panels in S1 and S2 Figs showing the measured values for actual ITD in μs (ITD vs ITD), actual ITD expressed as IPD in cycles (IPD vs ITD) and actual ILD in dB (ILD vs ITD) in relation to nominal ITD in μs and the measured values for the actual ILD in dB (ILD vs ILD), actual ITD expressed as IPD in cycles (IPD vs ILD) and actual ITD in μs (ITD vs ILD) in relation to nominal ILD in dB. Measured values are also given as error values (in μs, cycles, or dB) and relative errors (in %) for the worksheets ITD vs ITD, IPD vs ITD, and ILD vs ILD.
(XLSX)

S2 Table Binaural cues during free-field stimulation. Three gerbil carcasses (0, 1, 2) were measured in up to four different head positions (1, 2, 3, 4).
(XLSX)

S3 Table Virtual-headphone ITD thresholds. Six gerbils' ITD thresholds in μs were measured under virtual-headphone condition at frequencies between 750 and 2900 Hz. '—' indicates that an individual was not tested for a particular condition. 'x' indicates that no threshold could be determined for a particular condition.
(XLSX)

S4 Table Free-field ITD thresholds. Six gerbils' ITD thresholds in μs were derived from their free-field sound localization performance for frequencies between 750 and 3000 Hz. '—' indicates that an individual was not tested for a particular condition. Thresholds marked with * were derived from cumulative normal distribution functions that yielded R^2^<0.875; they were excluded from the calculation of the mean ITD thresholds.
(XLSX)

S5 Table Percentage ‘right’ response of four animals (Az, Sm, Lz, Gn) in response to ITDs larger than naturally occurring ITD in the gerbil. (XLSX)

S6 Table Virtual-headphone ILD thresholds. Six gerbils' ILD thresholds in dB were measured under virtual-headphone condition at the frequencies 2000, 4000, and 6000 Hz. '—' indicates that an individual was not tested for a particular condition.
(XLSX)

S7 Table Free-field ILD thresholds. Six gerbils' ILD thresholds in dB derived from their free-field sound localization performance for the frequencies 2000, 4000, and 6000 Hz. '—' indicates that an individual was not tested for a particular condition. Thresholds marked with * were derived from cumulative normal distribution functions that yielded R^2^<0.875; they were excluded from the calculation of the mean ILD thresholds.
(XLSX)
