# Supplementary figures and images for: Exploring binaural hearing in gerbils (Meriones unguiculatus) using virtual headphones
Source: PLoS One. 2017 Apr 10;12(4):e0175142. doi: 10.1371/journal.pone.0175142 (PMC5386270; doi:10.1371/journal.pone.0175142)

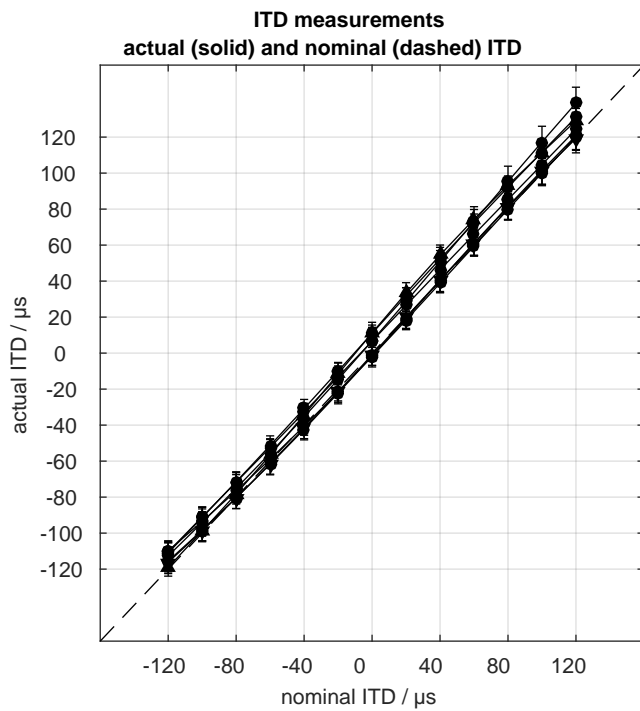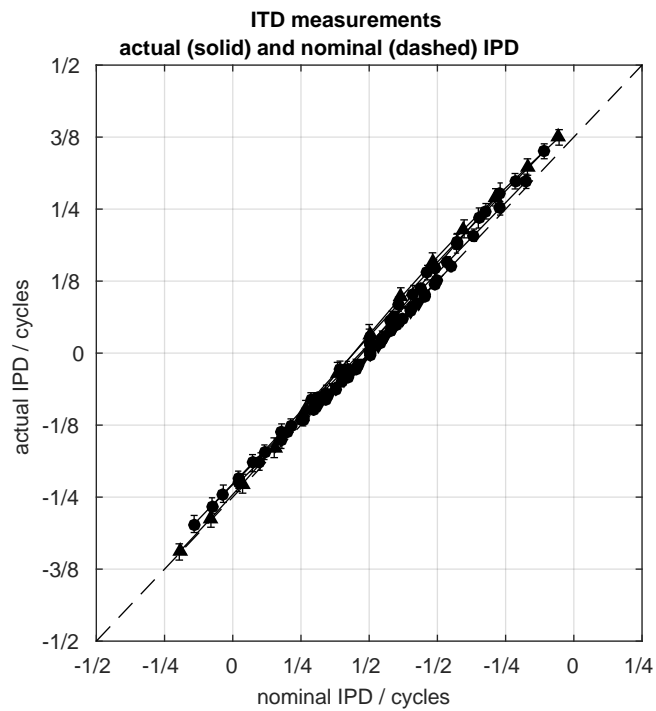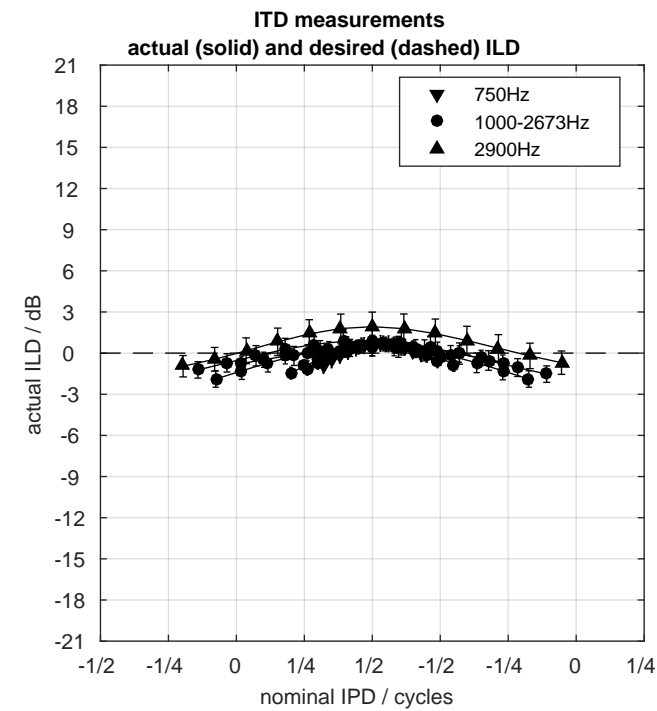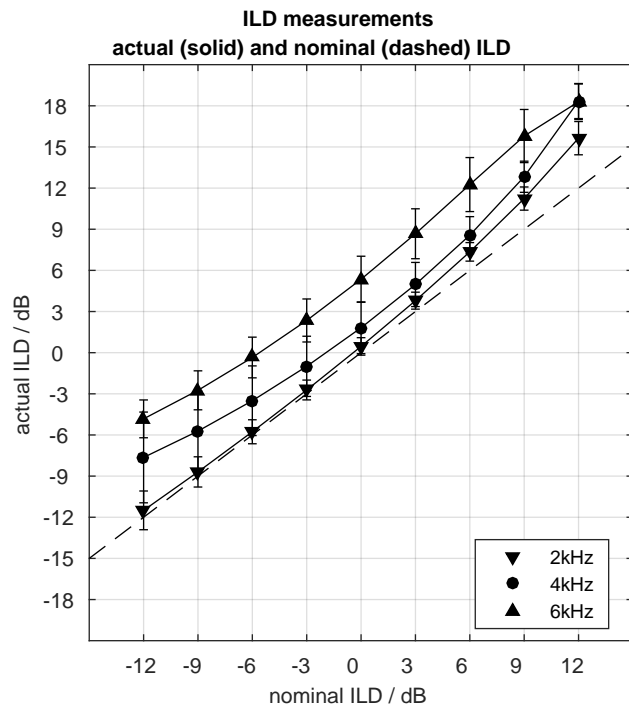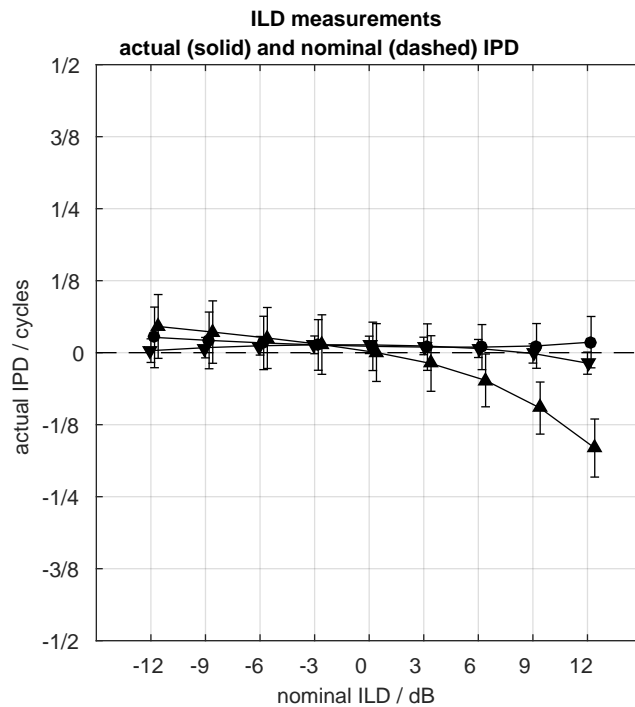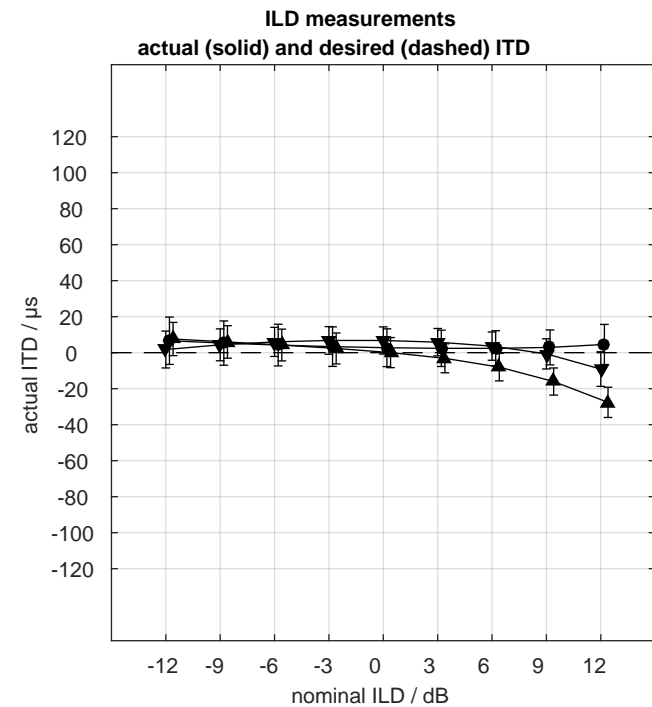

Supplement: S1 Fig — The upper row shows actual ITD (left), IPD (middle), and ILD (right) when presenting tones of different frequencies (0.75, 1, 1.25, 2, 2.4, 2.67, 2.9 kHz, lines) in relation to the nominal ITD (left) or the nominal ITD expressed as IPD (middle and right). The bottom row shows actual ILD (left), IPD (middle), and ITD (right) when presenting tones of different frequencies (2, 4, and 6 kHz, lines) in relation to the nominal ILD. Symbols and error bars indicate mean and standard deviation. Dashed lines indicate perfect cue reproduction. HRTFs between left and right speakers with both left and right ears, respectively, were measured with Etymotic ER-7C probe microphones from two gerbil carcasses in up to seven positions. The HRTFs were subsequently used as transfer function matrix H(ω) in Eq 3 with the original cross-talk cancellation matrix X(ω) (i.e., the same as in the experiments) to calculate the actual signals at the ears and to derive actual ILDs and ITDs with respect to the nominal binaural cues of the input signals in the simulations. (PDF) [file pone.0175142.s001.pdf]

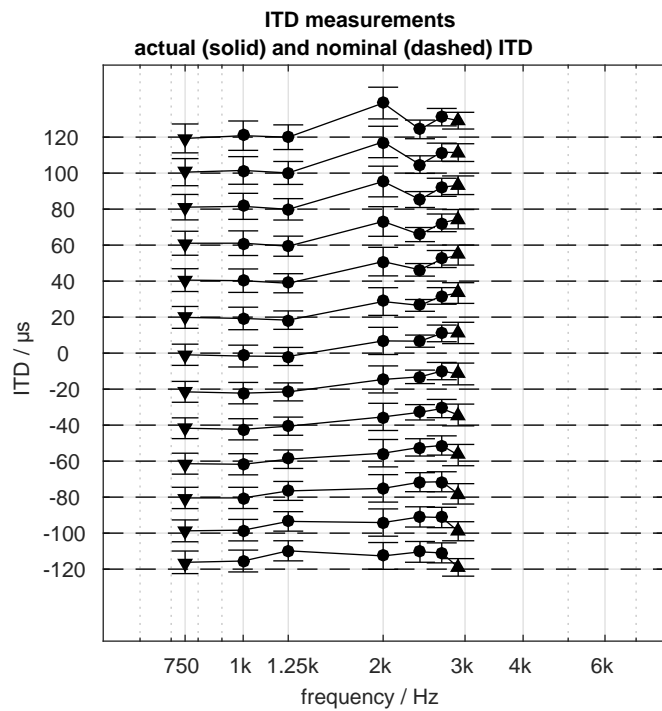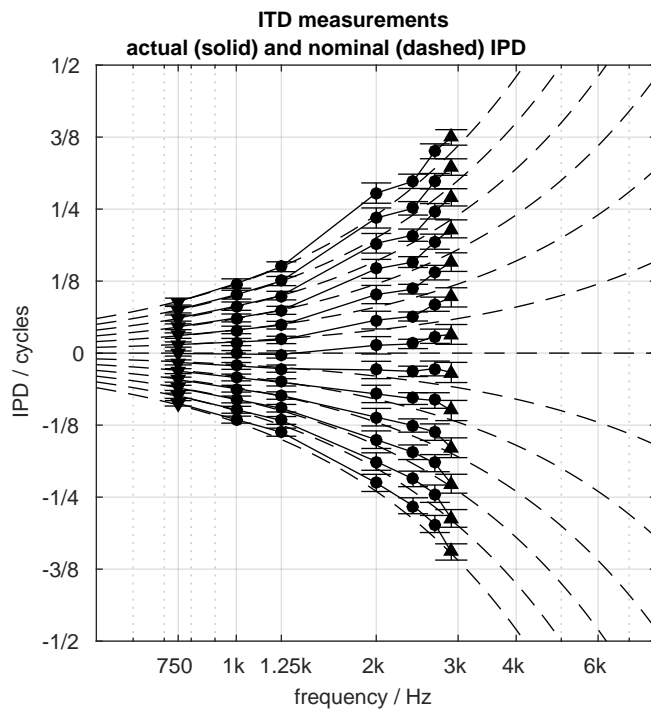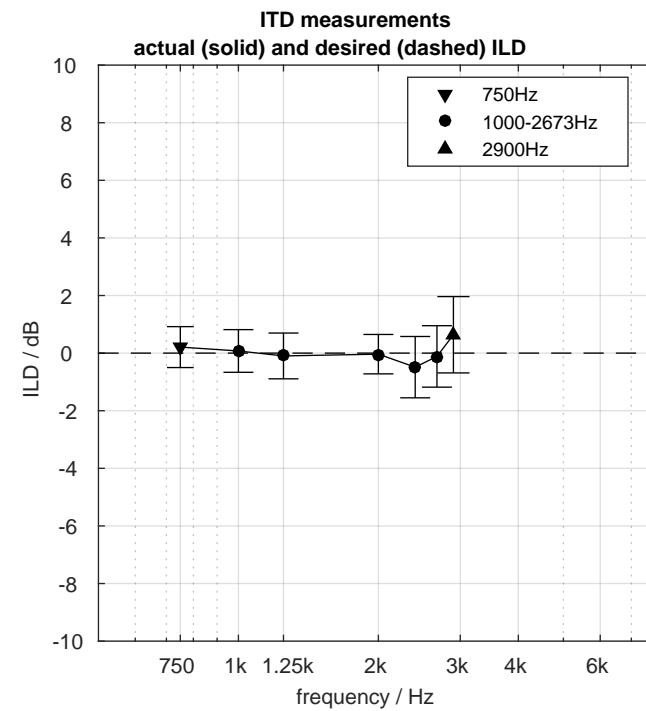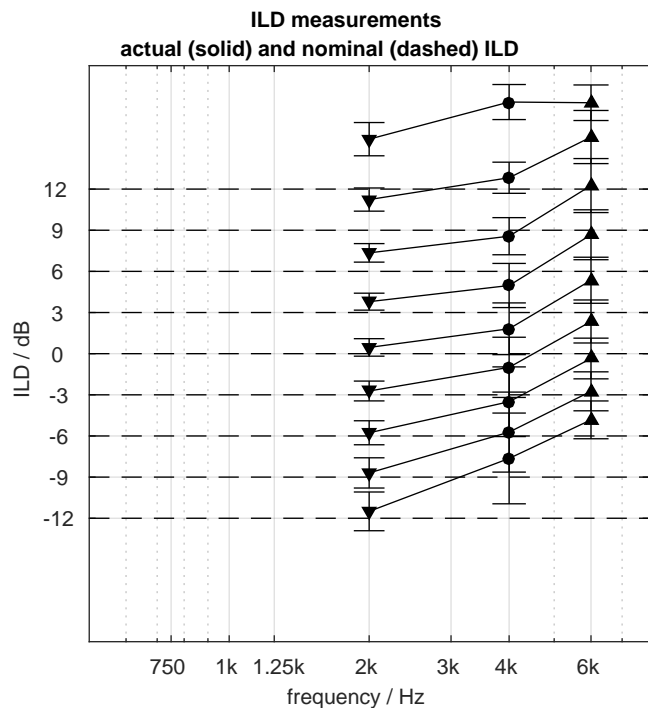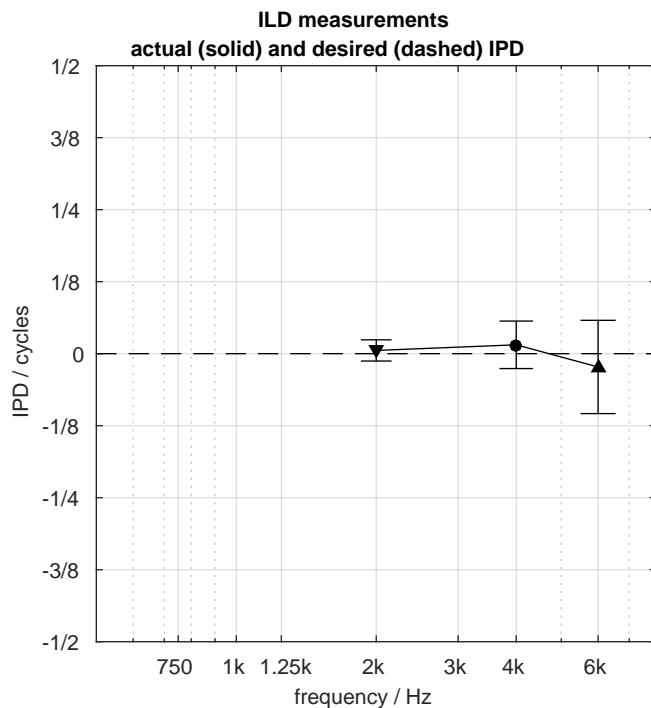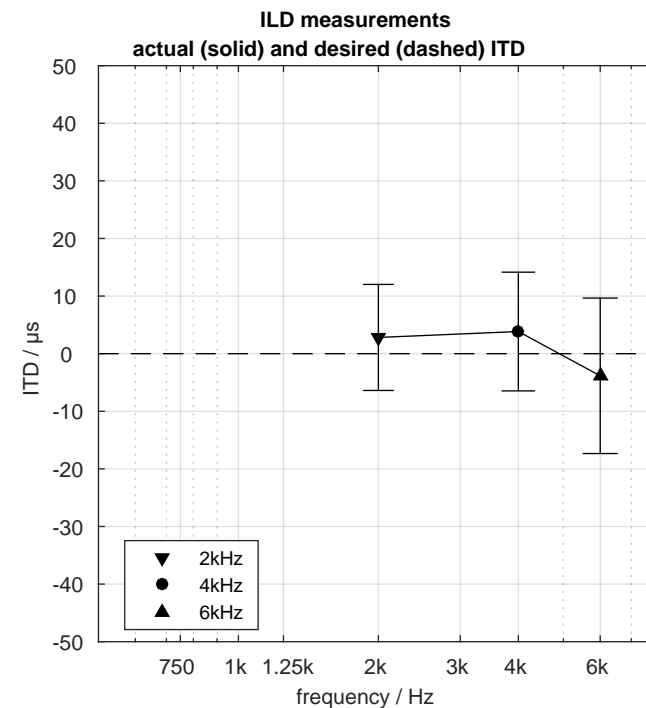

Supplement: S2 Fig — The upper row shows actual ITD (left), IPD (middle), and ILD (right) when tones of different frequency with an ITD were presented. The bottom row shows the actual ILD (left), IPD (middle), and ITD (right) when tones of different frequency with an ILD were presented. Symbols and error bars indicate mean and standard deviation. Dashed lines indicate perfect cue reproduction. For measurement and calculation details see S1 Fig. (PDF) [file pone.0175142.s002.pdf]

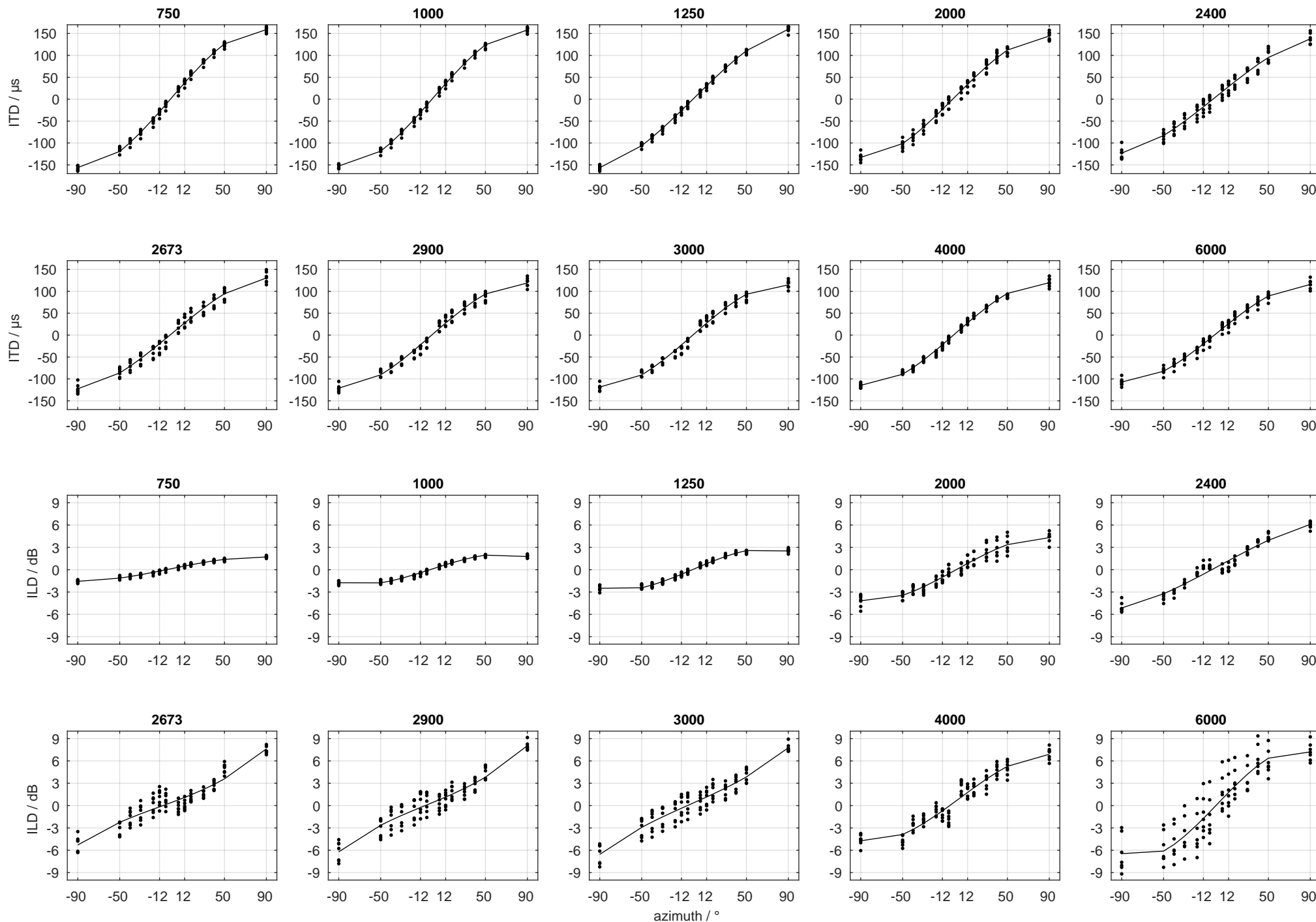

Supplement: S3 Fig — ITD (two top rows) and ILD (two bottom rows), measured for frequencies between 750 Hz and 6 kHz, change with the azimuthal position. ITD decrease slightly with increasing frequency, while ILD increase with increasing frequency. Symbols show the individual measurements from gerbil carcasses. The lines show the cubic polynomial fit used to calculate the ITDs and ILD for behavioral free-field measurements. (PDF) [file pone.0175142.s003.pdf]
